# Analysis of market equilibrium based on overconfidence behavior of market makers

**DOI:** 10.1371/journal.pone.0335569

**Published:** 2026-02-09

**Authors:** Ruohan Wang, Jing Wang, Zhi Yang

**Affiliations:** 1 College of Mathematics and Statistics, Yili Normal University, Yining, Xinjiang, China; 2 Institute of Applied Mathematics, Yili Normal University, Yining, Xinjiang, China; Minnan Normal University, CHINA

## Abstract

This paper constructs a theoretical model that includes heterogeneous risk-attitude insider traders (risk-neutral and risk-averse), overconfident market makers, and noise traders. It compares the model with two single-risk-attitude models. It systematically studies the impact mechanism of market makers’ overconfidence behavior on market equilibrium. The paper focuses on the influence of the degree of market makers’ overconfidence on market liquidity and the effect of heterogeneous risk attitudes on market stability. The study finds that in the heterogeneous risk-attitude insider trading model, market liquidity *λ* decreases within a specific range as the market maker’s confidence level *k* increases. Within another range, market liquidity *λ* increases as the confidence level *k* increases. Through comparative analysis of the three models, it is found that insider traders in the heterogeneous risk-attitude model can more actively use private information for trading, and their trading intensity and profit level are significantly higher than those of traders in the single-risk-attitude model. At the same time, the market’s residual information is less. This indicates that the aggressive strategy of risk-neutral insider traders and the conservative behavior of risk-averse insider traders form a complementary effect, which not only buffers the price impact of significant transactions but also promotes the efficient integration of information through diversified order flows. The numerical simulation results further confirm that market makers’ overconfidence will strengthen the information advantage of insider traders. In contrast, the differentiation of insider traders’ risk attitudes optimises the market’s information digestion mechanism, ultimately improving the efficiency of price formation.

## Introduction

Incorporating insights from traditional market microstructure theory, where insider trading disrupts market efficiency and market makers facilitate equilibrium through rational pricing, this study integrates behavioral finance perspectives highlighting pervasive cognitive biases among market participants. Barberis and Thaler [1]underscore the significance of psychological biases in asset pricing, with overconfidence emerging as a particularly salient deviation. Gervais and Odean [2] theoretically demonstrate that financial practitioners often overestimate their information-processing abilities. Extant literature has predominantly examined overconfidence from the investor’s viewpoint: Biais et al. [3] and Daniel et al. [4] posit that overconfident insider traders tend to overvalue the precision of private information, while Hirshleifer and Luo [5] suggest such behavior can amplify market volatility. However, existing research has paid insufficient attention to overconfidence among market makers, particularly lacking a systematic analysis of the interaction between overconfident market makers and insider traders with heterogeneous risk attitudes. To address this theoretical gap, this paper constructs a model that includes risk-neutral and risk-averse insider traders, an overconfident market maker, and noise traders. It focuses on examining the mechanisms through which the market maker’s level of confidence affects market liquidity, informational efficiency, and trading intensity, thereby deepening the understanding of the role of behavioral factors in market microstructure.

In Kyle [6] pioneering model, a single-period game-theoretic framework was established, including risk-neutral internal traders and market makers. This groundbreaking study introduced the linear pricing rule $\tilde{p}=\mu$  +  $\lambda \tilde{y}$, where the market maker determines the price based on the order flow $\tilde{y}$, where $\frac{1}{\lambda }$ (market depth) reflects the market maker’s assessment of the degree of information asymmetry, and *μ* represents the previous value of the asset. The market maker observes the total order flow to reverse-engineer internal information and ultimately achieves semi-strong form efficiency equilibrium - meaning that the market price can fully and instantaneously integrate all publicly available information. This model laid the theoretical foundation for market microstructure research and quantified the pricing rules of market makers as information intermediaries. In subsequent research, Kyle [7] introduced a new setting where market makers can observe public signals. This study demonstrated that when market makers use both the order flow and public signal $\tilde{s}$ for pricing $\tilde{p}=\mu \tilde{s}$  +  $\lambda \tilde{y}$, the phenomenon of information asymmetry is significantly reduced. The higher the accuracy of the public signal, the lower the reliance on the order flow (*λ* is smaller), and the market depth will correspondingly increase. This expansion marks the first time the pricing rules have been extended from a single information channel to a multi-dimensional information integration scenario.

Following this foundation, subsequent research has extended and deepened the Kyle model. Holden and Subrahmanyam [8] broke through the assumption of a single insider trader in the Kyle model by constructing a framework with N risk-neutral insider traders competing against each other. The study revealed that when multiple insider traders compete in the market, their competition accelerates the process of private information being incorporated into prices, resulting in market makers facing more intense order flow shocks. Market makers must adjust their pricing strategies to address this market structure change by increasing price sensitivity to order flow and widening bid-ask spreads. This ultimately causes market prices to converge more rapidly to efficient levels. Foster and Viswanathan [9] discovered that when insider traders possess varying levels of information precision, market makers must develop differentiated pricing strategies that assign greater weight to orders from traders with more accurate information, thereby maintaining pricing efficiency under conditions of market structure heterogeneity. Kyle and Wang [10] pioneered the introduction of the overconfidence assumption among market makers, demonstrating that they tend to underestimate market noise and adopt more aggressive pricing strategies. While this leads to pricing deviations, it may paradoxically result in higher order flow. Back [11] further extended this model to the continuous-time domain, demonstrating that the pricing coefficient λ(t) of market makers exhibits a dynamic decay characteristic during the process of information diffusion. The model aligns more with the fundamental markets’ gradual information disclosure process by introducing the time dimension. Viswanathan and Wang [12] conducted a systematic comparative study on the behavior of market makers in quote-driven markets and order-driven markets, providing an important theoretical basis for understanding the microstructural differences in modern securities markets.

In recent years, related research has continued to deepen. Cardenti and Stacetti [13] extended the Kyle model and incorporated random deadlines to reveal that market makers, when dynamically adjusting pricing rules due to information uncertainty, widen the bid-ask spread, thereby slowing down the speed at which information integrates into prices, and ultimately affecting market liquidity and equilibrium efficiency. Zhou [14] constructed an internal trading model that includes a risk-neutral internal trader and market makers who are overly confident in public signals. This study found that excessive confidence can enhance internal traders’ trading enthusiasm and profit level, improve market efficiency, and reduce the probability of unfavourable decisions. The research conducted by Ruan and Zhang [15] incorporated the factor of “investor attention", a limited rationality factor, into the model, providing a novel explanation for the phenomenon that public information has a significant impact on the market: Investors will reasonably allocate their limited attention to private information, resulting in insufficient attention to public information. Therefore, they must compensate for this by increasing the weight of public information in decision-making, ultimately exhibiting a “seemingly" excessive emphasis on public information in the market. On the other hand, Du and Zhu [16] conducted research on markets containing irrational noise traders. The behavioral deviations of these traders created more arbitrage opportunities for internal traders. They forced market makers to incorporate expectations of the behavioral patterns of noise traders into pricing, resulting in deviations between price differences and expected values. Jiang and Liu [17] discovered that those insider traders who possess internal information would distort their trading strategies due to the psychological bias of overconfidence, thereby significantly amplifying and accelerating the flow of internal information into the public domain, ultimately exacerbating the excessive reaction of the entire market to public information. Xiao et al. [18] extended the traditional model by introducing random deadlines and a partially observable optimal control framework, proving that even in a complex environment where insider traders face random trading time restrictions and have incomplete information, their overconfidence would prompt them to adopt more aggressive trading strategies, thereby accelerating the process of internal information integrating into the public price. Therefore, Daher [19] made two extensions based on the Zhou [14] model. The first extended model expanded the number of insider traders from a single entity to multiple insider traders, who formulated trading strategies based on the actual value of risky assets; the second extended model introduced multiple partially informed insider traders, who determined their trading strategies based on public signals. For each of the extended models, the research detailed the corresponding Nash equilibria and deeply analysed the results in the equilibrium state, including the trading strategies of insider traders, the degree of information disclosure, and changes in profits. Luo et al. [20] argued that overconfident traders would engage in more frequent trading based on noise, additional noise risk into asset prices. This priced in risk ultimately led to lower market risk-free interest rates and higher stock risk premiums. Daher and Damrah [21] demonstrated that when private signals are correlated with public signals, the overconfident mindset of internal traders (overestimating the accuracy of their private information) significantly enhances their trading enthusiasm and distorts their profits. This causes their private information to be incorporated into asset prices faster, but ultimately undermines their expected returns. While Daher and Damrah [21] explored multiple insiders, they did not consider the critical dimension of heterogeneous risk attitudes. Our paper bridges this gap by analyzing how the interaction between risk-heterogeneous insiders and an overconfident market maker alters market equilibrium.

Based on Kyle [6] theoretical framework, this paper constructs three types of insider trading models with different risk characteristics: the first type is a heterogeneous risk attitude model that includes risk-neutral insider traders, risk-averse insider traders, overconfident market makers, and noise traders; the second type is a homogeneous risk-neutral model that includes two risk-neutral insider traders, overconfident market makers, and noise traders; the third type is a homogeneous risk-averse model that includes two risk-averse insider traders, overconfident market makers, and noise traders. Firstly, this paper rigorously proves that these three models have a unique Nash equilibrium, and analyses and solves the key parameters in the equilibrium state, including the optimal trading intensity of insider traders and market liquidity, etc. To deeply explore the market equilibrium formation mechanism under the interaction of overconfident market makers and heterogeneous insider traders, this paper focuses on the impact of market makers’ confidence level on market liquidity. The research finds that within a specific range, market liquidity increases with the increase in confidence level, and in another range, it decreases with the increase in confidence level. Further, through numerical simulation, the differences in market performance of the three models in the equilibrium state are systematically compared. The results show that, compared with the two single risk attitude insider trading models, in the heterogeneous risk attitude insider trading model, due to the complementary strategies of different types of insider traders, insider traders are more actively using information for trading, resulting in more profits and less remaining information, and a more stable market.

The structure of this paper is as follows: In the second part, a heterogeneous risk attitude insider trading model is constructed, proving the existence of a unique Nash equilibrium, solving for the parameters that characterize the equilibrium, and examining the impact of the market maker’s overconfidence level on market liquidity. In the third part, two types of single risk attitude insider trading models are constructed, proving the existence of a unique Nash equilibrium and solving for the parameters that characterize the equilibrium. In the fourth part, a numerical model is presented to analyze key indicators such as the trading intensity, profits, and residual information volume of insider traders in the equilibrium state for the three types of models. In the fifth part, the conclusions of this paper are presented.

## Heterogeneous risk attitude model of insider trading

### The model

Based on Kyle’s (1985) one-period trading model, we assume that there are four types of traders in the market: a risk-neutral insider trader, a risk-averse insider trader, an overconfident market maker, and a noise trader. The liquidation value of tradable risky assets in the market is a random variable $\tilde{v}$, which follows a normal distribution with the mean of 0 and the variance of $\sigma_{v}^{2}$, that is, $\tilde{v}\sim N(0,\sigma_{v}^{2})$. $\tilde{v}$ is private information, and its true value is only known to the insider trader. $\tilde{s}=\tilde{v}+\tilde{\epsilon }$, this is the common information, which is known to both internal traders and market makers. Here, $\tilde{\epsilon }$ follows a normal distribution with the mean of 0 and the variance of $\sigma_{\epsilon }^{2}$, and it is independent of $\tilde{v}$. ${\sigma_{\epsilon }}^{-1}$ represents the accuracy of the common information. Internal traders can accurately estimate it, while overly confident market makers will overestimate the accuracy of the public signal, with the value of ${\left(k\sigma_{\epsilon }\right)}^{-1}$ (k≤1) [14]. Specifically, when *k* = 1, the market maker holds a rational belief about the public signal; when *k* < 1, the market maker underestimates the noise variance $Var(\tilde{\epsilon })$ in the public signal, thereby overestimating its precision (i.e., perceiving the precision as $(k\sigma_{\epsilon }{)}^{-1}>(\sigma_{\epsilon }{)}^{-1}$). In other words, internal traders hold a rational belief in the public signal: $\tilde{s}=\tilde{v}+\tilde{\epsilon }$. In contrast, overly confident market makers hold an overly high belief in the public signal: $\tilde{s}=\tilde{v}$  +  $k\tilde{\epsilon }$. This formula quantifies the weighting bias on private information through coefficient *k*, concisely capturing in a linear form the core behavioral characteristic of investors under overconfidence: deviating from rational expectations due to overvaluing their private information.

In the transaction, risk-neutral insider traders choose the trading volume $x_{1}(\tilde{s},\tilde{v})$ by maximising the expected return. At the same time, risk-averse insider traders choose their trading volume $x_{2}(\tilde{s},\tilde{v})$ by maximising the expected utility function. The utility function of the risk-averse insider traders is: *U*(*W*) = −*e*^−*AW*^, where A ≥0 is the risk aversion coefficient. The trading volume $\tilde{u}$ of the noise trader follows a normal distribution with the mean of 0 and the variance of $\sigma_{u}^{2}$, that is, $\tilde{u}\sim N(0,\sigma_{u}^{2})$. Suppose that the random variables $\tilde{v}$ and $\tilde{\epsilon }$ are independent of $\tilde{u}$. The market maker determines the price. *P*=$P(\tilde{s},\tilde{y})$ of the risky asset in a semi-strong efficient manner based on the total order flow $\tilde{y}=x_{1}(\tilde{s},\tilde{z})+x_{2}(\tilde{s},\tilde{z})+\tilde{u}$ and the public information $\tilde{s}$. It should be noted that the market maker cannot observe the individual values of $x_{1}(\tilde{s},\tilde{z})$, $x_{2}(\tilde{s},\tilde{z})$ and $\tilde{u}$.

Let $\pi_{1}$ represent the profit of a risk-neutral insider trader, ${\tilde{\pi }}_{1}=(\tilde{v}-P)x_{1}(\tilde{s},\tilde{v})$, and ${\tilde{\pi }}_{2}$ represent the profit of a risk-averse insider trader, $\pi_{2}=(\tilde{v}-P)x_{2}(\tilde{s},\tilde{v})$. *E*_*k*_ represents the conditional expectation under $\tilde{s}=\tilde{v}+k\tilde{\epsilon }$, and *E* represents the conditional expectation under $\tilde{s}=\tilde{v}+\tilde{\epsilon }$.

**Definition 1.** The equilibrium formed by the trading strategies of risk-neutral and risk-averse insiders, as well as the pricing rules of market makers, satisfies the following relationship:

(1) Maximization of Profit: For risk-neutral insider traders, any other trading strategy $x_{1}^{\prime }$,

$\text{E}[(\tilde{v}-P(x_{1}(\tilde{s},\tilde{v})+x_{2}(\tilde{s},\tilde{v})+\tilde{u}))x_{1}(\tilde{s},\tilde{v})]$ $\geq \text{E}[(\tilde{v}-P(x_{1}^{\prime }(\tilde{s},\tilde{v})+x_{2}(\tilde{s},\tilde{v})+\tilde{u}))x_{1}^{\prime }(\tilde{s},\tilde{v})]$.

For risk-averse insider traders, any other trading strategy $x_{2}^{\prime }$,


$$\text{E}[-e^{-A(\tilde{v}-P(x_{1}(\tilde{s},\tilde{v})+x_{2}(\tilde{s},\tilde{v})+\tilde{u}))x_{2}(\tilde{s},\tilde{v})}]\;\geq \text{E}[-e^{-A(\tilde{v}-P(x_{1}(\tilde{s},\tilde{v})+x_{2}^{\prime }(\tilde{s},\tilde{v})+\tilde{u}))x_{2}^{\prime }(\tilde{s},\tilde{v})}].$$

(2) Market efficiency:


$$P(\tilde{s},\tilde{y})=E_{k}[\tilde{v}|\tilde{s},\tilde{y}].$$

### The only linear equalization

We are concerned about the linear Nash equilibrium to avoid the difficulties arising from the involvement of higher-order expectations.

**Proposition 1.** *The unique Nash equilibrium expression is:*

$$x_{1}(\tilde{s},\tilde{v})=\alpha_{0}\tilde{v}+\alpha_{1}\tilde{s},$$ (1)

$$x_{2}(\tilde{s},\tilde{v})=\beta_{0}\tilde{v}+\beta_{1}\tilde{s},$$ (2)

$$P(\tilde{s},\tilde{y})=\lambda \tilde{y}+\mu \tilde{s}\text{.}$$ (3)

*Where*

$$\alpha_{0}=\frac{A\lambda \sigma_{u}^{2}+1}{3\lambda +2A\lambda^{2}\sigma_{u}^{2}},$$ (4)

$$\alpha_{1}=\frac{-(A\lambda \sigma_{u}^{2}+1)\mu }{3\lambda +2A\lambda^{2}\sigma_{u}^{2}},$$ (5)

$$\beta_{0}=\frac{1}{3\lambda +2A\lambda^{2}\sigma_{u}^{2}},$$ (6)

$$\beta_{1}=\frac{-\mu }{3\lambda +2A\lambda^{2}\sigma_{u}^{2}},$$ (7)

$$\mu =\frac{\sigma_{v}^{2}}{\sigma_{v}^{2}+k^{2}\sigma_{\epsilon }^{2}},$$ (8)

*the parameter λ satisfies:*

$$4A^{2}\sigma_{u}^{6}\sigma_{v}^{2}\lambda^{4}+4k^{2}A^{2}\sigma_{u}^{6}\sigma_{\epsilon }^{2}\lambda^{4}+12A\sigma_{u}^{4}\sigma_{v}^{2}\lambda^{3}+12k^{2}A\sigma_{u}^{4}\sigma_{\epsilon }^{2}\lambda^{3}+9\sigma_{u}^{2}\sigma_{v}^{2}\lambda^{2}\;+9k^{2}\sigma_{u}^{2}\sigma_{\epsilon }^{2}\lambda^{2}-k^{2}A^{2}\sigma_{u}^{4}\sigma_{v}^{2}\sigma_{\epsilon }^{2}\lambda^{2}-3k^{2}A\sigma_{u}^{2}\sigma_{v}^{2}\sigma_{\epsilon }^{2}\lambda -2k^{2}\sigma_{v}^{2}\sigma_{\epsilon }^{2}=0\text{.}$$ (9)

*Profit of risk-neutral insider traders*

$$E[\pi_{1}]=(\alpha_{0}+\alpha_{1}-\alpha_{0}\mu -\alpha_{1}\mu -\lambda \alpha_{0}^{2}-2\lambda \alpha_{0}\alpha_{1}-\lambda \alpha_{1}^{2}-\lambda \alpha_{0}\beta_{0}\;-\lambda \alpha_{1}\beta_{0}-\lambda \alpha_{0}\beta_{1}-\lambda \alpha_{1}\beta_{1})\sigma_{v}^{2}-(\alpha_{1}\mu +\lambda \alpha_{1}^{2}+\lambda \alpha_{1}\beta_{1})\sigma_{\epsilon }^{2}.$$ (10)

*Profit of risk-averse insider traders*

$$E[\pi_{2}]=(\beta_{0}+\beta_{1}-\beta_{0}\mu -\beta_{1}\mu -\lambda \alpha_{0}\beta_{0}-\lambda \alpha_{0}\beta_{1}-\lambda \alpha_{1}\beta_{0}\;-\lambda \alpha_{1}\beta_{1}-\lambda \beta_{0}^{2}-2\lambda \beta_{0}\beta_{1}-\lambda \beta_{1}^{2})\sigma_{v}^{2}-(\beta_{1}\mu +\lambda \alpha_{1}\beta_{1}+\lambda \beta_{1}^{2})\sigma_{\epsilon }^{2}.$$ (11)

*Remaining information quantity*

$$\Sigma =\frac{\sigma_{v}^{2}\sigma_{\epsilon }^{2}\sigma_{u}^{2}}{(\alpha_{0}+\beta_{0}{)}^{2}\sigma_{v}^{2}\sigma_{\epsilon }^{2}+(\sigma_{v}^{2}+\sigma_{\epsilon }^{2})\sigma_{u}^{2}}.$$ (12)

***Proof:*** See The Appendix A.1. □

From Eqs (4) to (7), it can be seen that $\alpha_{0}$ and $\beta_{0}$ are greater than zero, while $\alpha_{1}$ and $\beta_{1}$ are less than zero. This indicates that risk-neutral and risk-averse insider traders attach significant weight to private information and weaken the role of public information. This trading behavior that utilises public information provides a cover for insider traders to conduct transactions based on private information.

According to Proposition 1, we obtain the following conclusion:

**Proposition 2.**


$$\frac{d\lambda }{dk}\left\{ \begin{array}{cc} <0, & \lambda \in (0,\lambda_{1})\cup (x_{0},\frac{k\sigma_{v}\sigma_{\epsilon }}{2\sigma_{u}\sqrt{\sigma_{v}^{2}+k^{2}\sigma_{\epsilon }^{2}}})\\ >0, & \lambda \in (\lambda_{1},x_{0}) \end{array}\right.,$$

Among them, $\lambda_{1}=\frac{-6S+\sqrt{36S^{2}+32A^{2}\sigma_{u}^{2}k^{2}\sigma_{v}^{2}\sigma_{\varepsilon }^{2}S}}{16A\sigma_{u}^{2}S}$, and *x*_0_ is a solution to a quartic polynomial equation in *λ*.

Proposition 2 reveals a dual effect of overconfidence in market microstructure. When the market maker is highly overconfident (small *k*), they significantly underestimate noise in the public signal and tend to overadjust prices in response to order flow. This amplifies adverse selection risk and reduces liquidity. As *k* increases toward rationality, the market maker’s misjudgment diminishes, leading to more stable price adjustments and improved liquidity. However, when *k* approaches 1 (the rational benchmark), complete reliance on public information may reduce sensitivity to private information, thereby affecting liquidity formation once again. This non-monotonic relationship suggests that moderate confidence helps strike a balance between informational sensitivity and pricing stability, whereas extreme behavioral biases can disrupt price discovery and liquidity provision through distinct channels.

***Proof:*** See The Appendix A.2. □

## Two types of insider trading models with single-risk attitude

By applying the equilibrium condition defined , the parameters in the model with only two risk-neutral insider traders in the market and the model with only two risk-averse insider traders in the market are calculated, respectively.

### The unique linear equilibrium under the single risk neutrality insider trading model

**Proposition 3.** *The unique Nash equilibrium expression is:*

$$x_{1}(\tilde{s},\tilde{v})=\alpha_{0}^{N}\tilde{v}+\alpha_{1}^{N}\tilde{s},$$ (13)

$$x_{2}(\tilde{s},\tilde{v})=\beta_{0}^{N}\tilde{v}+\beta_{1}^{N}\tilde{s},$$ (14)

$$P(\tilde{s},\tilde{y})=\lambda^{N}\tilde{y}+\mu^{N}\tilde{s}\text{.}$$ (15)

*Where*

$$\alpha_{0}^{N}=\beta_{0}^{N}=\frac{\sigma_{u}\sqrt{\sigma_{v}^{2}+k^{2}\sigma_{\varepsilon }^{2}}}{2k\sigma_{v}\sigma_{\varepsilon }},$$ (16)

$$\alpha_{1}=\beta_{1}^{N}=\frac{-\sigma_{u}\sigma_{v}}{2k\sigma_{\varepsilon }\sqrt{\sigma_{v}^{2}+k^{2}\sigma_{\varepsilon }^{2}}},$$ (17)

$$\mu^{N}=\frac{\sigma_{v}^{2}}{\sigma_{v}^{2}+k^{2}\sigma_{\epsilon }^{2}},$$ (18)

$$\lambda^{N}=\frac{k\sigma_{v}\sigma_{\varepsilon }}{2\sigma_{u}\sqrt{\sigma_{v}^{2}+k^{2}\sigma_{\varepsilon }^{2}}}.$$ (19)

*Profit of risk-neutral insider traders*

$$E[\pi^{N}]=(\alpha_{0}^{N}+\alpha_{1}^{N}-\alpha_{0}^{N}\mu^{N}-\alpha_{1}^{N}\mu^{N}-2\lambda^{N}(\alpha_{0}^{N}{)}^{2}-4\lambda^{N}\alpha_{0}^{N}\alpha_{1}^{N}-\;2\lambda^{N}(\alpha_{1}^{N}{)}^{2})\sigma_{v}^{2}-(\alpha_{1}^{N}\mu^{N}+2\lambda^{N}(\alpha_{1}^{N}{)}^{2})\sigma_{\epsilon }^{2}.$$ (20)

*Remaining information quantity*

$$\Sigma^{N}=\frac{\sigma_{v}^{2}\sigma_{\epsilon }^{2}}{\frac{\sigma_{v}^{2}}{k}+\sigma_{v}^{2}+2\sigma_{\epsilon }^{2}}.$$ (21)

***Proof:*** See The Appendix A.3. □

### The unique Linear Equilibrium under the Model of Single Risk-Averse Insider Trading

**Proposition 4.** *The unique Nash equilibrium expression is:*

$$x_{1}(\tilde{s},\tilde{v})=\alpha_{0}^{A}\tilde{v}+\alpha_{1}^{A}\tilde{s},$$ (22)

$$x_{2}(\tilde{s},\tilde{v})=\beta_{0}^{A}\tilde{v}+\beta_{1}^{A}\tilde{s},$$ (23)

$$P(\tilde{s},\tilde{y})=\lambda^{A}\tilde{y}+\mu^{A}\tilde{s}\text{.}$$ (24)

*Where*

$$\alpha_{0}^{A}=\beta_{0}^{A}=\frac{1}{4\lambda^{A}+A(\lambda^{A}{)}^{2}\sigma_{u}^{2}},$$ (25)

$$\alpha_{1}^{A}=\beta_{1}^{A}=\frac{-\mu^{A}}{4\lambda^{A}+A(\lambda^{A}{)}^{2}\sigma_{u}^{2}},$$ (26)

$$\mu^{A}=\frac{\sigma_{v}^{2}}{\sigma_{v}^{2}+k^{2}\sigma_{\epsilon }^{2}},$$ (27)

*the parameter $\lambda^{A}$ satisfies:*

$$A^{2}\sigma_{u}^{6}\sigma_{v}^{2}(\lambda^{A}{)}^{4}+A^{2}k^{2}\sigma_{u}^{2}\sigma_{\varepsilon }^{2}(\lambda^{A}{)}^{4}+8A\sigma_{u}^{4}\sigma_{v}^{2}{\left(\lambda^{A}\right)}^{3}+8Ak^{2}\sigma_{u}^{4}\sigma_{\varepsilon }^{2}{\left(\lambda^{A}\right)}^{3}\;+16\sigma_{u}^{2}\sigma_{v}^{2}{\left(\lambda^{A}\right)}^{2}+16k^{2}\sigma_{u}^{2}\sigma_{\varepsilon }^{2}{\left(\lambda^{A}\right)}^{2}-2Ak^{2}\sigma_{u}^{2}\sigma_{v}^{2}\sigma_{\varepsilon }^{2}\lambda^{A}-4k^{2}\sigma_{v}^{2}\sigma_{\varepsilon }^{2}=0\text{.}$$ (28)

*Profit of risk-averse insider traders*

$$E[\pi^{A}]=(\alpha_{0}^{A}+\alpha_{1}^{A}-\alpha_{0}^{A}\mu^{A}-\alpha_{1}^{A}\mu^{A}-2\lambda^{A}(\alpha_{0}^{A}{)}^{2}-4\lambda^{A}\alpha_{0}^{A}\alpha_{1}^{A}\;-2\lambda^{A}(\alpha_{1}^{A}{)}^{2})\sigma_{v}^{2}-(\alpha_{1}^{A}\mu^{A}+2\lambda^{A}(\alpha_{1}^{A}{)}^{2})\sigma_{\epsilon }^{2}.$$ (29)

*Remaining information quantity*

$$\Sigma^{A}=\frac{\sigma_{v}^{2}\sigma_{\epsilon }^{2}\sigma_{u}^{2}}{({2\alpha }_{0}^{A}{)}^{2}\sigma_{v}^{2}\sigma_{\epsilon }^{2}+(\sigma_{v}^{2}+\sigma_{\epsilon }^{2})\sigma_{u}^{2}}.$$ (30)

***Proof:*** See The Appendix A.4. □

## Numerical analysis

Next, the characteristics of the linear equilibrium of the three types of models are analysed and compared from the perspectives of the trading intensity, profits, and price effectiveness of insider traders.

### Insider traders’ trading intensity

Under the three types of models, the trading intensity of insider traders is measured by $\alpha_{0}$, $\beta_{0}$, $\alpha_{0}^{N}$, and $\beta_{0}^{N}$. With $\sigma_{u}=1$, $\sigma_{v}=1$, $\sigma_{\epsilon }=1$, and *A* = 1, next, by selecting different *k* values, the trading intensities under the three types of models are calculated respectively, as shown in Table 1.

**Table 1:** Three types of model trading intensity.

*α*/*k*	0.2	0.3	0.4	0.5	0.6	0.7	0.8	0.9	1.0
$\alpha_{0}$	3.81	2.67	2.06	1.76	1.55	1.43	1.33	1.28	1.20
$\beta_{0}$	3.49	2.36	1.76	1.47	1.26	1.14	1.05	1.00	0.93
$\alpha_{0}^{N}$	2.55	1.73	1.35	1.12	0.98	0.87	0.80	0.75	0.71
$\alpha_{0}^{A}$	2.44	1.72	1.25	1.08	0.90	0.80	0.75	0.70	0.66

From Table 1, it can be clearly seen that the relationship in terms of the trading intensity of insider traders under the three types of models is as follows


$$\alpha_{0}>\beta_{0}>\alpha_{0}^{N}>\alpha_{0}^{A}.$$

Based on the data in Table 1, the curves of the trading intensity of insider traders with respect to *k* under the three types of models were simultaneously plotted. This enabled a more intuitive comparison and analysis of the variation characteristics of the trading intensity of insider traders in different models.

**Fig 1:**
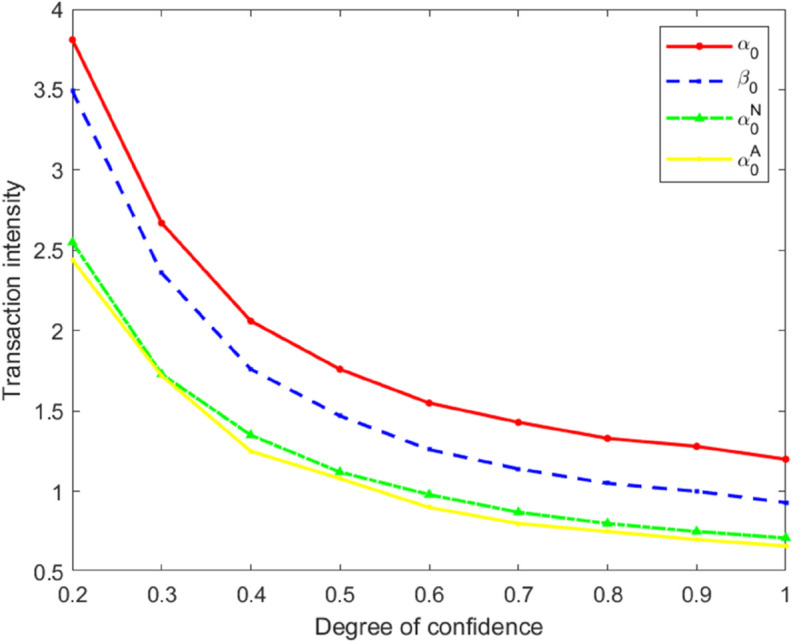
Comparison chart of transaction intensity under different *k* values.

Fig 1 shows that the trading intensity of insider traders decreases as the market maker’s confidence level *k* increases. This indicates that an overconfident market maker, by overestimating the precision of public information, responds less sensitively to private information in pricing, thereby creating greater informational advantage for insider traders and incentivizing them to trade more aggressively based on private information. This is consistent with the conclusion of Zhou [14].

When comparing trading activity across the three models, we find that the overall trading activity in the heterogeneous risk-attitude model is significantly higher than in the single risk-attitude models. This is primarily due to a trading-shielding effect created by the presence of risk-averse insider traders for their risk-neutral counterparts. Risk-averse traders reduce their trading volume to avoid uncertainty; their conservative strategy effectively absorbs part of the market impact and slows price adjustment to order flow, thereby reducing the overall information sensitivity of the market. Within this “buffered” environment, risk-neutral traders can execute larger trades at a relatively lower price-impact cost, allowing them to exploit private information more fully for arbitrage. The dynamic between the two types of traders forms a strategic complementarity—the caution of risk-averse traders creates room for action by risk-neutral traders, while the active trading of risk-neutral traders enhances the efficiency and stealth of information incorporation into prices. In contrast, in the single risk-attitude models, all traders exhibit similar risk responses, making the market’s interpretation of order flow more concentrated and sensitive. This causes private information to be absorbed into prices more rapidly, forcing insider traders to reduce trading intensity to avoid premature exposure. This mechanism demonstrates that heterogeneity in risk attitudes, by constructing an endogenous trading buffer and information-shielding structure, significantly shapes the path of information transmission and the formation of liquidity in the market, thereby deepening our understanding of behavioral interaction and efficiency formation in market microstructure.

### Profits of insider traders

Under the three types of models, the profits of insider traders are measured by $E[\pi_{1}]$, $E[\pi_{2}]$, $E[\pi^{N}]$, and $E[\pi^{A}]$. Next, by selecting different values of *k*, the profits of insider traders under the three types of models are calculated, respectively, as shown in Table 2.

**Table 2:** Profit of the three types of insider traders.

*π*/*k*	0.2	0.3	0.4	0.5	0.6	0.7	0.8	0.9	1.0
$E[\pi_{1}]$	2.74	1.89	1.38	1.10	0.91	0.76	0.65	0.57	0.51
$E[\pi_{2}]$	1.10	0.70	0.47	0.35	0.27	0.23	0.20	0.18	0.17
$E[\pi^{N}]$	1.15	0.76	0.55	0.39	0.30	0.24	0.21	0.19	0.18
$E[\pi^{A}]$	1.15	0.76	0.50	0.39	0.29	0.24	0.21	0.19	0.18

From Table 2, it can be clearly seen that the profit levels of insider traders under the three types of models are as follows:


$$E[\pi_{1}]>E[\pi^{N}]>E[\pi^{A}]>E[\pi_{2}].$$

Based on the data in Table 2, the curves of insider traders’ profits varying with *k* under the three types of models were simultaneously plotted. This enabled a more intuitive comparison and analysis of the profit variation characteristics of insider traders in different models.

**Fig 2:**
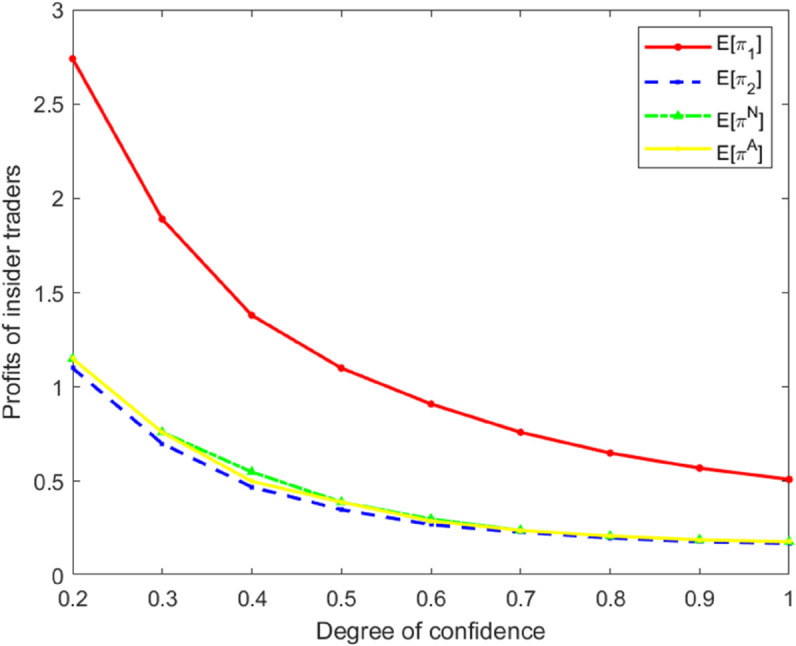
Profit comparison chart for different *k* values.

As shown in Fig 2, the trading profits of insider traders are inversely proportional to the market maker’s degree of overconfidence *k*. This indicates that market maker overconfidence significantly enhances the profit opportunities for insider traders, consistent with the findings of Zhou [14]. A comparison of profit levels across the three models further reveals that the ordering of profits aligns with the earlier conclusions regarding trading intensity. Notably, in the heterogeneous risk-attitude model, the profits of risk-neutral insider traders are substantially higher than those in the other two models. This suggests that when risk-averse traders adopt conservative strategies due to risk avoidance, the overall price adjustment slows. In such an environment, the liquidity provision by risk-neutral insider traders effectively reduces transaction execution costs, enabling them to exploit private information more efficiently for large-scale arbitrage. This result not only highlights the substantial impact of risk-attitude heterogeneity on informational efficiency and profit distribution but also provides new micro-level evidence on how different trader behaviors jointly shape market performance and arbitrage opportunities.

### Informational efficiency

Under the three types of models, the remaining information quantity of the transaction is measured by Σ, $\Sigma^{N}$, and $\Sigma^{A}$, respectively. Set $\sigma_{u}=1$, $\sigma_{v}=1$, $\sigma_{\epsilon }=1$, and *A* = 1. Then, different *k* values are selected, and the remaining information quantity is calculated under the three types of models, respectively. See Table 3.

**Table 3:** The remaining information content of the three types of models.

Σ/*k*	0.2	0.3	0.4	0.5	0.6	0.7	0.8	0.9	1.0
Σ	0.01	0.02	0.03	0.04	0.05	0.06	0.07	0.08	0.10
$\Sigma^{N}$	0.13	0.16	0.18	0.20	0.21	0.22	0.23	0.24	0.25
$\Sigma^{A}$	0.13	0.22	0.32	0.36	0.41	0.44	0.45	0.47	0.48

From Table 3, it can be clearly seen that the relationship in terms of the amount of remaining information under the three types of models is as follows:


$$\Sigma^{A}>\Sigma^{N}>\Sigma .$$

Based on the data in Table 3, the curves of the remaining information quantity varying with *k* under the three types of models were simultaneously plotted. This enabled a more intuitive comparison and analysis of the variation characteristics of the remaining information quantity in different models.

**Fig 3:**
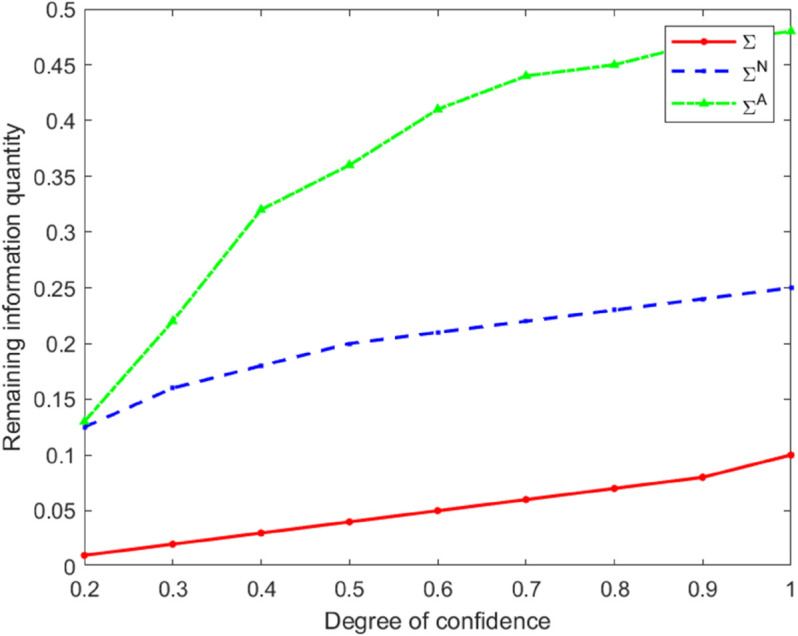
Comparison chart of remaining information quantity under different *k* values.

Fig 3 shows that the remaining information quantity is an increasing function of the degree of overconfidence *k*, which implies that overconfidence among market makers will create a more efficient market, which is consistent with the conclusion of Zhou [14]. A further comparison of residual information across the three models reveals that the heterogeneous risk-attitude model exhibits the lowest residual information. This indicates that insider traders in this model more actively exploit their informational advantages. The divergence in market risk attitudes reduces the price impact of aggressive trading: risk-averse insider traders curtail competitive trading out of caution, while risk-neutral insider traders provide liquidity, thereby facilitating more efficient incorporation of information into prices. In contrast, in the single risk-averse model, insider traders behave more conservatively due to concerns about exposure risk, which prevents the market from fully digesting private information and results in higher residual information. Collectively, these findings demonstrate that heterogeneity in traders’ risk attitudes significantly enhances informational efficiency by influencing trading behavior and the information-aggregation mechanism. While market-maker overconfidence to some extent amplifies insider profits, it also strengthens the market’s overall capacity to absorb information. This provides a new perspective for understanding the complex interplay among behavioral biases, market structure, and informational efficiency.

## Conclusion

This study constructs a theoretical model of insider traders with heterogeneous risk attitudes and overconfident market makers to systematically analyze the complex interaction between behavioral factors and market structure. The main findings show that market liquidity is non-monotonically affected by the degree of overconfidence of market makers, and risk attitude heterogeneity plays a key role in this. Specifically, there is significant strategic complementarity among insiders with different risk preferences: risk-averse traders absorb price shocks through conservative strategies to create liquidity buffers for the market; Risk-neutral traders use this buffer to trade more actively, thus improving the efficiency of information integration. Compared with the single risk attitude model, this endogenous complementary mechanism makes the heterogeneous risk attitude model show better performance in terms of trading activity, profitability and information efficiency, highlighting the significance of the diversity of participants’ behaviors for market resilience.

The above research results show that a resilient market depends not only on the rationality of the participants, but also on the interaction structure composed of their risk preferences and the diversity of behavioral patterns. The effective operation of this structure puts forward specific requirements for the design of market system: it needs the regulatory framework to identify and maintain this endogenous complementary relationship and avoid the damage caused by the homogenization policy; Market microstructure is also needed to support the effective participation of diversified investors, so that each type of investor can play its role according to its characteristics.

At the level of regulatory practice, regulators should first recognize the endogenous stabilizing function of market participants’ risk heterogeneity and incorporate it into the policy evaluation dimension. Excessive homogenization of regulatory requirements may destroy the natural risk stratification and buffering mechanism of the market, but aggravate the vulnerability of the system. Therefore, while ensuring fairness and transparency, the regulatory framework should strive to maintain a complementary structure among investors with different risk appetities. For example, risk-neutral traders who function as liquidity providers can be appropriately flexible in trading restrictions to promote price discovery; As the cornerstone of market stability, risk-averse long-term investors should strengthen their risk management requirements. At the same time, regulators should pay close attention to the behavioral deviations of intermediary institutions such as market makers, and prevent pricing distortion and liquidity sudden changes caused by overconfidence through the combination of stress testing and behavioral supervision.

In summary, this study not only provides a new theoretical perspective for understanding the role of behavioral heterogeneity in market microstructure, but also provides a theoretical basis for building an institutional framework that takes into account both efficiency and stability in realistic heterogeneous markets.

## Appendix

### Appendix A.1. Proof of Proposition 1

***Proof:*** The following proof adopts the backwards recursion method. Let the insider trader’s trading strategy and the market maker’s pricing rules be linear functions.


$$x_{1}(\tilde{s},\tilde{v})=\alpha_{0}\tilde{v}+\alpha_{1}\tilde{s},x_{2}(\tilde{s},\tilde{v})=\beta_{0}\tilde{v}+\beta_{1}\tilde{s}\text{,,}P(\tilde{s},\tilde{y})=\lambda \tilde{y}+\mu \tilde{s},$$

where, $\alpha_{0}$, $\alpha_{1}$, $\beta_{0}$ and $\beta_{1}$ are all constants.

Risk-neutral insider traders formulate their trading strategies based on maximising expected profits.


$$E[(\tilde{v}-P)x_{1}(\tilde{v},\tilde{s})|\tilde{v},\tilde{s}]=E[(\tilde{v}-\lambda \tilde{y}-\mu \tilde{s})x_{1}(\tilde{v},\tilde{s})|\tilde{v},\tilde{s}]\;=E[(\tilde{v}-\lambda (x_{1}(\tilde{v},\tilde{s})+x_{2}(\tilde{v},\tilde{s})+\tilde{u})-\mu \tilde{s})x_{1}(\tilde{v},\tilde{s})|\tilde{v},\tilde{s}]\;=E[(\tilde{v}-\lambda x_{1}(\tilde{v},\tilde{s})-\lambda (\beta_{0}\tilde{v}+\beta_{1}\tilde{s})-\mu \tilde{s})x_{1}(\tilde{v},\tilde{s})|\tilde{v},\tilde{s}]-E[(-\lambda \tilde{u})x_{1}(\tilde{v},\tilde{s})|\tilde{v},\tilde{s}]\;=(\tilde{v}-\lambda x_{1}(\tilde{v},\tilde{s})-\lambda (\beta_{0}\tilde{v}+\beta_{1}\tilde{s})-\mu \tilde{s})x_{1}(\tilde{v},\tilde{s})+\lambda x_{1}(\tilde{v},\tilde{s})E[\tilde{u}]\;=(\tilde{v}-\lambda x_{1}(\tilde{v},\tilde{s})-\lambda (\beta_{0}\tilde{v}+\beta_{1}\tilde{s})-\mu \tilde{s})x_{1}(\tilde{v},\tilde{s}).$$

Taking the first derivative of the above equation and setting it equal to zero, we obtain


$$(\tilde{v}-\lambda x_{1}(\tilde{v},\tilde{s})-\lambda (\beta_{0}\tilde{v}+\beta_{1}\tilde{s})-\mu {\tilde{s}}_{k})-\lambda x_{1}(\tilde{v},\tilde{s})=0.$$

This can be rearranged to give


$$x_{1}(\tilde{v},\tilde{s})=\frac{1}{2\lambda }(1-\beta_{0}\lambda )\tilde{v}-\frac{1}{2\lambda }(-\beta_{1}\lambda -\mu )\tilde{s}.$$

Therefore

$$\alpha_{0}=\frac{(1-\beta_{0}\lambda )\tilde{v}}{2\lambda },$$ (A1)

$$\alpha_{1}=\frac{-\beta_{1}\lambda -\mu }{2\lambda }.$$ (A2)

Risk-averse insider traders obtain their trading strategies by maximising the expected value of the negative utility index.


$$E[-e^{-A(\tilde{v}-P)x_{2}(\tilde{v},\tilde{s})}|\tilde{s},\tilde{v}]\;=E[-e^{-A(\tilde{v}-\lambda \tilde{y}-\mu \tilde{s})x_{2}(\tilde{v},\tilde{s})}|\tilde{s},\tilde{v}]\;=-e^{-A(\tilde{v}-\lambda (\alpha_{0}\tilde{v}+\alpha_{1}\tilde{s})-\lambda x_{2}(\tilde{v},\tilde{s})-\mu \tilde{s})x_{2}(\tilde{v},\tilde{s})}\cdot E[-e^{-A(-\lambda \tilde{u})x_{2}(\tilde{v},\tilde{s})}|\tilde{s},\tilde{v}]\;=-e^{-A(\tilde{v}-\lambda (\alpha_{0}\tilde{v}+\alpha_{1}\tilde{s})-\lambda x_{2}(\tilde{v},\tilde{s})-\mu \tilde{s})x_{2}(\tilde{v},\tilde{s})}\cdot (-e^{\frac{A^{2}\lambda^{2}x_{2}(\tilde{v},\tilde{s}{)}^{2}{\sigma_{u}}^{2}}{2}})\;=-e^{-A(\tilde{v}-\lambda (\alpha_{0}\tilde{v}+\alpha_{1}\tilde{s})-\lambda x_{2}(\tilde{v},\tilde{s})-\mu \tilde{s})x_{2}(\tilde{v},\tilde{s})+\frac{A^{2}\lambda^{2}{x_{2}(\tilde{v},\tilde{s})}^{2}{\sigma_{u}}^{2}}{2}}.$$

Taking the first derivative of the above equation and setting it equal to zero, we obtain


$$-e^{-A(\tilde{v}-\lambda (\alpha_{0}\tilde{v}+\alpha_{1}\tilde{s})-\lambda x_{2}(\tilde{v},\tilde{s})-\mu \tilde{s})x_{2}(\tilde{v},\tilde{s})+\frac{A^{2}\lambda^{2}x_{2}(\tilde{v},\tilde{s}{)}^{2}\sigma_{u}^{2}}{2}}\;\cdot (-A(\tilde{v}-\lambda (\alpha_{0}\tilde{v}+\alpha_{1}\tilde{s})-\lambda x_{2}(\tilde{v},\tilde{s})-\mu \tilde{s})+A\lambda x_{2}(\tilde{v},\tilde{s})+A^{2}\lambda^{2}\sigma_{u}^{2}x_{2}(\tilde{v},\tilde{s}))=0.$$

This can be rearranged to give


$$x_{2}(\tilde{v},\tilde{s})=\frac{A-A\lambda \alpha_{0}}{2A\lambda +A^{2}\lambda^{2}\sigma_{u}^{2}}\tilde{v}+\frac{-A\lambda \alpha_{1}-A\mu }{2A\lambda +A^{2}\lambda^{2}\sigma_{u}^{2}}\tilde{s}.$$

Therefore

$$\beta_{0}=\frac{A-A\lambda \alpha_{0}}{2A\lambda +A^{2}\lambda^{2}\sigma_{u}^{2}},$$ (A3)

$$\beta_{1}=\frac{-A\lambda \alpha_{1}-A\mu }{2A\lambda +A^{2}\lambda^{2}\sigma_{u}^{2}}.$$ (A4)

From Eq (31), we have $2\lambda \alpha_{0}=1-\beta_{0}\lambda$. Therefore, $\beta_{0}=\frac{1-2\lambda \alpha_{0}}{\lambda }$. Combining this with Eq (33), we obtain


$$\alpha_{0}=\frac{A\lambda \sigma_{u}^{2}+1}{3\lambda +2A\lambda^{2}\sigma_{u}^{2}}.$$

Substitute (*A*1) into (*A*3), we obtain


$$\beta_{0}=\frac{1}{3\lambda +2A\lambda^{2}\sigma_{u}^{2}}.$$

From Eq (32), we have $2\lambda \alpha_{1}=\beta_{1}\lambda -\mu$. Therefore, $\beta_{1}=-\frac{2\lambda \alpha_{1}+\mu }{\lambda }$. Combining this with Eq (34), we obtain


$$\alpha_{1}=\frac{(-A\lambda \sigma_{u}^{2}-1)\mu }{3\lambda +2A\lambda^{2}\sigma_{u}^{2}}.$$

Substituting Eq (32) into Eq (34), we obtain


$$\beta_{1}=\frac{-\mu }{3\lambda +2A\lambda^{2}\sigma_{u}^{2}}.$$

Based on semi-strong efficiency and Lemma 1 and 2 in Zhou [14], we know that $\tilde{v}-E_{k}(\tilde{v}|\tilde{s})$ is independent of $\tilde{s}$ under the belief $\tilde{s}=\tilde{v}+k\tilde{\epsilon }$, and meanwhile


$$\sigma \{(\alpha_{0}+\beta_{0})\tilde{v}+(\alpha_{1}+\beta_{1})\tilde{s}+\tilde{u},\tilde{s}\}=\sigma \{(\alpha_{0}+\beta_{0})(\tilde{v}-E_{k}(\tilde{v}|\tilde{s}))+\tilde{u},\tilde{s}\}.\;\text{Then}$$

$$P(\tilde{s},\tilde{v})=E_{k}[\tilde{v}|\tilde{s},\tilde{y}]=E_{k}[\tilde{v}-E_{k}(\tilde{v}|\tilde{s})+E_{k}(\tilde{v}|\tilde{s})|x_{1}(\tilde{v},\tilde{s})+x_{2}(\tilde{v},\tilde{s})+\tilde{u},\tilde{s}]\;=E_{k}[\tilde{v}-E_{k}(\tilde{v}|\tilde{s})|x_{1}(\tilde{v},\tilde{s})+x_{2}(\tilde{v},\tilde{s})+\tilde{u},\tilde{s}]\;+E_{k}[E_{k}(\tilde{v}|\tilde{s})|x_{1}(\tilde{v},\tilde{s})+x_{2}(\tilde{v},\tilde{s})+\tilde{u},\tilde{s}]\;=E_{k}[\tilde{v}-E_{k}(\tilde{v}|\tilde{s})|(\alpha_{0}+\beta_{0})(\tilde{v}-E_{k}(\tilde{v}|\tilde{s}))+\tilde{u}]+E_{k}(\tilde{v}|\tilde{s})\;=E[\tilde{v}-E_{k}(\tilde{v}|\tilde{s})]+\frac{\mathrm{cov}(\tilde{v}-E_{k}(\tilde{v}|\tilde{s}),(\alpha_{0}+\beta_{0})(\tilde{v}-E_{k}(\tilde{v}|\tilde{s}))+\tilde{u})}{\mathrm{var}((\alpha_{0}+\beta_{0})(\tilde{v}-E_{k}(\tilde{v}|\tilde{s}))+\tilde{u})}\cdot \;((\alpha_{0}+\beta_{0})(\tilde{v}-E_{k}(\tilde{v}|\tilde{s}))+\tilde{u})-E[(\alpha_{0}+\beta_{0})(\tilde{v}-E_{k}(\tilde{v}|\tilde{s}))+\tilde{u}]+E_{k}(\tilde{v}|\tilde{s})\;=\frac{(\alpha_{0}+\beta_{0})H}{(\alpha_{0}+\beta_{0}{)}^{2}H+\sigma_{u}^{2}}((\alpha_{0}+\beta_{0})[\tilde{v}-E_{k}(\tilde{v}|\tilde{s})]+\tilde{u})+c_{k}\tilde{s}\;=\frac{(\alpha_{0}+\beta_{0})H}{(\alpha_{0}+\beta_{0}{)}^{2}H+\sigma_{u}^{2}}\tilde{y}+\frac{(\alpha_{0}+\beta_{0})H}{(\alpha_{0}+\beta_{0}{)}^{2}H+\sigma_{u}^{2}}[-(\alpha_{0}+\beta_{0})c_{k}-(\alpha_{1}+\beta_{1})+c_{k}]\tilde{s}.$$

Where

$$c_{k}=\frac{\sigma_{v}^{2}}{\sigma_{v}^{2}+k^{2}\sigma_{\epsilon }^{2}},$$ (A5)

$$H=Var(\tilde{v}-E_{k}(\tilde{v}-\tilde{s}))=E_{k}(\tilde{v}-E_{k}(\tilde{v}|\tilde{s}){)}^{2}=(1-c_{k}{)}^{2}\sigma_{v}^{2}+k^{2}c_{k}^{2}\sigma_{\epsilon }^{2}=\frac{k^{2}\sigma_{v}^{2}\sigma_{\epsilon }^{2}}{\sigma_{v}^{2}+k^{2}\sigma_{\epsilon }^{2}}.$$ (A6)

Therefore

$$\mu =\frac{(\alpha_{0}+\beta_{0})H}{(\alpha_{0}+\beta_{0}{)}^{2}H+\sigma_{u}^{2}}[-(\alpha_{0}+\beta_{0})c_{k}-(\alpha_{1}+\beta_{1})+c_{k}],$$ (A7)

$$\lambda =\frac{(\alpha_{0}+\beta_{0})H}{(\alpha_{0}+\beta_{0}{)}^{2}H+\sigma_{u}^{2}}.$$ (A8)

Substituting Eqs (4) to (7) into Eq (37) yields


$$\mu =c_{k}=\frac{\sigma_{v}^{2}}{\sigma_{v}^{2}+k^{2}\sigma_{\epsilon }^{2}}.$$

Substituting (4) and (5) into (*A*8) and then simplifying, we can obtain a quartic equation involving *λ*.


$$4A^{2}\sigma_{u}^{6}\sigma_{v}^{2}\lambda^{4}+4k^{2}A^{2}\sigma_{u}^{6}\sigma_{\epsilon }^{2}\lambda^{4}+12A\sigma_{u}^{4}\sigma_{v}^{2}\lambda^{3}+12k^{2}A\sigma_{u}^{4}\sigma_{\epsilon }^{2}\lambda^{3}+9\sigma_{u}^{2}\sigma_{v}^{2}\lambda^{2}\;+9k^{2}\sigma_{u}^{2}\sigma_{\epsilon }^{2}\lambda^{2}-k^{2}A^{2}\sigma_{u}^{4}\sigma_{v}^{2}\sigma_{\epsilon }^{2}\lambda^{2}-3k^{2}A\sigma_{u}^{2}\sigma_{v}^{2}\sigma_{\epsilon }^{2}\lambda -2k^{2}\sigma_{v}^{2}\sigma_{\epsilon }^{2}=0\text{.}\;E[\pi_{1}]=E[(\tilde{v}-P)\;x_{1}(\tilde{v},\tilde{s})]=E[E(\tilde{v}-P)x_{1}(\tilde{v},\tilde{s})|\tilde{v},\tilde{s}]\;=E[E[(\tilde{v}-\lambda \tilde{y}-\mu \tilde{s})(\alpha_{0}\tilde{v}+\alpha_{1}\tilde{s})|\tilde{v},\tilde{s}]]\;=E[(\tilde{v}-\mu \tilde{s})(\alpha_{0}\tilde{v}+\alpha_{1}\tilde{s})-\lambda (\alpha_{0}\tilde{v}+\alpha_{1}\tilde{s}+\beta_{0}\tilde{v}+\beta_{1}\tilde{s})(\alpha_{0}\tilde{v}+\alpha_{1}\tilde{s})]\;=E[\alpha_{0}{\tilde{v}}^{2}+\alpha_{1}\tilde{v}(\tilde{v}+\tilde{\epsilon })-\alpha_{0}\mu \tilde{v}(\tilde{v}+\tilde{\epsilon })-\alpha_{1}\mu (\tilde{v}+\tilde{\epsilon }{)}^{2}\;-\lambda (\alpha_{0}^{2}{\tilde{v}}^{2}+\alpha_{0}\alpha_{1}\tilde{v}(\tilde{v}+\tilde{\epsilon })+\alpha_{0}\alpha_{1}\tilde{v}(\tilde{v}+\tilde{\epsilon }))\;-\lambda (\alpha_{1}^{2}(\tilde{v}+\tilde{\epsilon }{)}^{2}+\alpha_{0}\beta_{0}{\tilde{v}}^{2}+\alpha_{1}\beta_{0}\tilde{v}(\tilde{v}+\tilde{\epsilon })+\alpha_{0}\beta_{1}\tilde{v}(\tilde{v}+\tilde{\epsilon })+\alpha_{1}\beta_{1}(\tilde{v}+\tilde{\epsilon }{)}^{2})]\;=(\alpha_{0}+\alpha_{1}-\alpha_{0}\mu -\alpha_{1}\mu -\lambda \alpha_{0}^{2}-2\lambda \alpha_{0}\alpha_{1}-\lambda \alpha_{1}^{2}-\lambda \alpha_{0}\beta_{0}-\lambda \alpha_{1}\beta_{0})\sigma_{v}^{2}\;-(\lambda \alpha_{0}\beta_{1}+\lambda \alpha_{1}\beta_{1})\sigma_{v}^{2}-(\alpha_{1}\mu +\lambda \alpha_{1}^{2}+\lambda \alpha_{1}\beta_{1})\sigma_{\epsilon }^{2}.\;E[\pi_{2}]=E[(\tilde{v}-P)\;x_{2}(\tilde{v},\tilde{s})]=E[E(\tilde{v}-P)x_{2}(\tilde{v},\tilde{s})|\tilde{v},\tilde{s}]\;=E[E[(\tilde{v}-\lambda \tilde{y}-\mu \tilde{s})(\beta_{0}\tilde{v}+\beta_{1}\tilde{s})|\tilde{v},\tilde{s}]]\;=E[(\tilde{v}-\mu \tilde{s})(\beta_{0}\tilde{v}+\beta_{1}\tilde{s})-\lambda (\alpha_{0}\tilde{v}+\alpha_{1}\tilde{s}+\beta_{0}\tilde{v}+\beta_{1}\tilde{s})(\beta_{0}\tilde{v}+\beta_{1}\tilde{s})]\;=E[\beta_{0}{\tilde{v}}^{2}+\beta_{1}\tilde{v}(\tilde{v}+\tilde{\epsilon })-\beta_{0}\mu \tilde{v}(\tilde{v}+\tilde{\epsilon })-\beta_{1}\mu (\tilde{v}+\tilde{\epsilon }{)}^{2}\;-\lambda (\alpha_{0}\beta_{0}{\tilde{v}}^{2}+\alpha_{0}\beta_{1}\tilde{v}(\tilde{v}+\tilde{\epsilon })+\alpha_{1}\beta_{0}\tilde{v}(\tilde{v}+\tilde{\epsilon }))\;-\lambda (\alpha_{1}\beta_{1}(\tilde{v}+\tilde{\epsilon }{)}^{2}+\beta_{0}^{2}{\tilde{v}}^{2}+\beta_{0}\beta_{1}\tilde{v}(\tilde{v}+\tilde{\epsilon })+\beta_{0}\beta_{1}\tilde{v}(\tilde{v}+\tilde{\epsilon })+\beta_{1}^{2}(\tilde{v}+\tilde{\epsilon }{)}^{2})]\;=(\beta_{0}+\beta_{1}-\beta_{0}\mu -\beta_{1}\mu -\lambda \alpha_{0}\beta_{0}-\lambda \alpha_{0}\beta_{1}-\lambda \alpha_{1}\beta_{0}-\lambda \alpha_{1}\beta_{1})\sigma_{v}^{2}\;-(\lambda \beta_{0}^{2}+2\lambda \beta_{0}\beta_{1}+\lambda \beta_{1}^{2})\sigma_{v}^{2}-(\beta_{1}\mu +\lambda \alpha_{1}\beta_{1}+\lambda \beta_{1}^{2})\sigma_{\epsilon }^{2}.$$

The equation regarding the parameter Σ is


$$\Sigma =\mathrm{Var}(\tilde{v}|P,\tilde{s})=\mathrm{Var}(\tilde{v}-E(\tilde{v}|\tilde{s})|\lambda \tilde{y}+\mu \tilde{s},\tilde{s})\;=\mathrm{Var}(\tilde{v}-E(\tilde{v}|\tilde{s})|x_{1}(\tilde{v},\tilde{s})+x_{2}(\tilde{v},\tilde{s})+\tilde{u},\tilde{s})\;=\mathrm{Var}(\tilde{v}-E(\tilde{v}|\tilde{s})|(\alpha_{0}+\beta_{0})(\tilde{v}-E(\tilde{v}|\tilde{s}))+\tilde{u})\;=\mathrm{Var}(\tilde{v}-E(\tilde{v}|\tilde{s}))-\frac{\mathrm{cov}(\tilde{v}-E(\tilde{v}|\tilde{s}),(\alpha_{0}+\beta_{0})(\tilde{v}-E(\tilde{v}|\tilde{s}))+\tilde{u}{)}^{2}}{\mathrm{var}((\alpha_{0}+\beta_{0})(\tilde{v}-E(\tilde{v}|\tilde{s}))+\tilde{u})}\;=E(\tilde{v}-E(\tilde{v}|\tilde{s}){)}^{2}-\frac{(\alpha_{0}+\beta_{0}{)}^{2}\mathrm{Var}(\tilde{v}-E(\tilde{v}|\tilde{s}){)}^{2}}{(\alpha_{0}+\beta_{0}{)}^{2}\mathrm{Var}(\tilde{v}-E(\tilde{v}|\tilde{s}))+\sigma_{u}^{2}}\;=\frac{E(\tilde{v}-E(\tilde{v}|\tilde{s}){)}^{2}\sigma_{u}^{2}}{(\alpha_{0}+\beta_{0}{)}^{2}E(\tilde{v}-E(\tilde{v}|\tilde{s}){)}^{2}+\sigma_{u}^{2}}\;=\frac{\sigma_{v}^{2}\sigma_{\epsilon }^{2}\sigma_{u}^{2}}{(\alpha_{0}+\beta_{0}{)}^{2}\sigma_{v}^{2}\sigma_{\epsilon }^{2}+(\sigma_{v}^{2}+\sigma_{\epsilon }^{2})\sigma_{u}^{2}}.$$

□

### Appendix A.2. Proof of Proposition 2

***Proof:*** Let

$$f(\lambda ,k)=4A^{2}\sigma_{u}^{6}\sigma_{v}^{2}\lambda^{4}+4k^{2}A^{2}\sigma_{u}^{6}\sigma_{\epsilon }^{2}\lambda^{4}+12A\sigma_{u}^{4}\sigma_{v}^{2}\lambda^{3}+12k^{2}A\sigma_{u}^{4}\sigma_{\epsilon }^{2}\lambda^{3}+9\sigma_{u}^{2}\sigma_{v}^{2}\lambda^{2}\;+9k^{2}\sigma_{u}^{2}\sigma_{\epsilon }^{2}\lambda^{2}-k^{2}A^{2}\sigma_{u}^{4}\sigma_{v}^{2}\sigma_{\epsilon }^{2}\lambda^{2}-3k^{2}A\sigma_{u}^{2}\sigma_{v}^{2}\sigma_{\epsilon }^{2}\lambda -2k^{2}\sigma_{v}^{2}\sigma_{\epsilon }^{2}=0.$$ (A9)

By rearranging Eq (39), we obtain:


$$[4\sigma_{u}^{2}(\sigma_{v}^{2}+k^{2}\sigma_{\varepsilon }^{2})\lambda^{2}-k^{2}\sigma_{v}^{2}\sigma_{\varepsilon }^{2}](A^{2}\sigma_{u}^{2}\lambda^{2}+3A\sigma_{u}^{2}\lambda +2)=-\sigma_{u}^{2}(\sigma_{v}^{2}+k^{2}\sigma_{\varepsilon }^{2})\lambda^{2}<0.$$

Then


$$4\sigma_{u}^{2}(\sigma_{u}^{2}+k^{2}\sigma_{\varepsilon }^{2})\lambda^{2}-k_{v}^{2}\sigma_{v}^{2}\sigma_{\epsilon }^{2}<0.$$

That is


$$\lambda <\frac{k\sigma_{v}\sigma_{\epsilon }}{2\sigma_{u}\sqrt{S}},\;where\;S=\sigma_{v}^{2}+k^{2}\sigma_{\epsilon }^{2}.$$

Substituting λ=0 and $\lambda =\frac{k\sigma_{v}\sigma_{\epsilon }}{2\sigma_{u}\sqrt{S}}$ into Eq (39), we have *f*(0,*k*) < 0; $f(\frac{k\sigma_{v}\sigma_{\epsilon }}{2\sigma_{u}\sqrt{S}},k)>0.$ Therefore, the root of f(λ,k)=0 lies in the interval $\left(0,\frac{k\sigma_{v}\sigma_{\epsilon }}{2\sigma_{u}\sqrt{S}}\right)$.

The relationship between *λ* and *k* is discussed below, where *A*, $\sigma_{u}$, $\sigma_{v}$, and $\sigma_{\epsilon }$ are treated as parameters, according


$$\frac{d\lambda }{dk}=-\frac{\frac{\partial f}{\partial k}}{\frac{\partial f}{\partial \lambda }},$$

where $\frac{\partial f}{\partial \lambda }$ is nonzero, we can obtain

$$\frac{d\lambda }{dk}=\frac{1}{\left(8A\sigma_{u}^{4}\sigma_{v}^{2}\lambda^{2}+8Ak^{2}\sigma_{u}^{4}\sigma_{\varepsilon }^{2}\lambda^{2}+6\sigma_{u}^{2}\sigma_{v}^{2}\lambda^{+}6k^{2}\sigma_{u}^{2}\sigma_{\varepsilon }^{2}\lambda -k^{2}A\sigma_{u}^{2}\sigma_{v}^{2}\sigma_{\varepsilon }^{2}\right)}\cdot \;\frac{-[(2A\sigma_{u}^{2}\lambda +4)(4kA\sigma_{u}^{4}\sigma_{\varepsilon }^{2}\lambda^{3}+4k\sigma_{u}^{2}\sigma_{\varepsilon }^{2}\lambda^{2}-kA\sigma_{u}^{2}\sigma_{v}^{2}\sigma_{\varepsilon }^{2}\lambda -k\sigma_{v}^{2}\sigma_{\varepsilon }^{2})+2k\sigma_{u}^{2}\sigma_{\varepsilon }^{2}\lambda^{2}]}{2A\sigma_{u}^{2}\lambda +3}.$$ (A10)

The following analyzes the sign of $\frac{d\lambda }{dk}$ over the interval $\left(0,\frac{k\sigma_{v}\sigma_{\epsilon }}{2\sigma_{u}\sqrt{S}}\right)$. First, consider the first term of Eq (40):

(i) For $8A\sigma_{u}^{4}\sigma_{v}^{2}\lambda^{2}+8Ak^{2}\sigma_{u}^{4}\sigma_{\varepsilon }^{2}\lambda^{2}+6\sigma_{u}^{2}\sigma_{v}^{2}\lambda^{+}6k^{2}\sigma_{u}^{2}\sigma_{\varepsilon }^{2}\lambda -k^{2}A\sigma_{u}^{2}\sigma_{v}^{2}\sigma_{\varepsilon }^{2}$, setting it equal to zero yields $\lambda_{1}=\frac{-6S+\sqrt{36S^{2}+32A^{2}\sigma_{u}^{2}k^{2}\sigma_{v}^{2}\sigma_{\varepsilon }^{2}S}}{16A\sigma_{u}^{2}S}$. Moreover, since $\frac{k\sigma_{v}\sigma_{\epsilon }}{2\sigma_{u}\sqrt{S}}>\lambda_{1}$ it follows that when $\lambda \in (0,\lambda_{1})$, this term is negative, while when$\lambda \in (\lambda_{1},\frac{k\sigma_{v}\sigma_{\epsilon }}{2\sigma_{u}\sqrt{S}})$, it is positive.

Next, let us analyse the second term of Eq (40).

(ii) Since the denominator in the second term is greater than zero, only the numerator needs to be analysed. Substitute λ=0 and $\lambda =\frac{k\sigma_{v}\sigma_{\epsilon }}{2\sigma_{u}\sqrt{S}}$ into the numerator, respectively, and calculate to obtain a value greater than zero and a value less than zero. Moreover, since the derivative of the numerator is less than zero between these two values, there must be a zero point between them, denoted as *x*_0_. Through calculation, it can be found that substituting $\lambda =\frac{2\sigma_{v}}{5\sigma_{u}}$ into the numerator results in a value greater than zero, while $\lambda_{1}<\frac{2\sigma_{v}}{5\sigma_{u}}$, thus $\lambda_{1}<x_{0}$. Therefore, when $\lambda \in (0,\lambda_{1})$, this term is greater than zero, when $\lambda \in (\lambda_{1},x_{0})$, this term is greater than zero, and when $\lambda \in (x_{0},\frac{k\sigma_{v}\sigma_{\epsilon }}{2\sigma_{u}\sqrt{S}})$, this term is less than zero.

Therefore, when $\lambda \in (0,\lambda_{1})\cup (x_{0},\frac{k\sigma_{v}\sigma_{\epsilon }}{2\sigma_{u}\sqrt{\sigma_{v}^{2}+k^{2}\sigma_{\epsilon }^{2}}})$, $\frac{d\lambda }{dk}<0$, and when $\lambda \in (\lambda_{1},x_{0})$, $\frac{d\lambda }{dk}>0$. □

### Appendix A.3. Proof of Proposition 3

***Proof:*** The partial proof process is briefly written since the calculation method is similar to Proposition 1. Let the insider trader’s trading strategy and the market maker’s pricing rules be linear functions.


$$x_{1}(\tilde{s},\tilde{v})=\alpha_{0}^{N}\tilde{v}+\alpha_{1}^{N}\tilde{s}=x_{2}(\tilde{s},\tilde{v})=\beta_{0}^{N}\tilde{v}+\beta_{1}^{N}\tilde{s},P(\tilde{s},\tilde{y})=\lambda^{N}\tilde{y}+\mu^{N}\tilde{s}\text{,}$$

where, $\alpha_{0}^{N}$, $\alpha_{1}^{N}$, $\beta_{0}^{N}$ and $\beta_{1}^{N}$ are all constants.

Risk-neutral insider traders formulate their trading strategies based on maximising expected profits.


$$E[(\tilde{v}-P)x_{1}(\tilde{v},\tilde{s})|\tilde{v},\tilde{s}]=E[(\tilde{v}-\lambda^{N}\tilde{y}-\mu^{N}\tilde{s})x_{1}(\tilde{v},\tilde{s})|\tilde{v},\tilde{s}]\;=E[(\tilde{v}-2\lambda^{N}x_{1}(\tilde{v},\tilde{s})-\lambda \tilde{u}-\mu^{N}\tilde{s})x_{1}(\tilde{v},\tilde{s})|\tilde{v},\tilde{s}]\;=(\tilde{v}-2\lambda^{N}x_{1}(\tilde{v},\tilde{s})-\mu^{N}\tilde{s})x_{1}(\tilde{v},\tilde{s}).$$

Taking the first derivative of the above equation and setting it equal to zero, we obtain


$$\tilde{v}-2\lambda^{N}x_{1}(\tilde{v},\tilde{s})-\mu^{N}\tilde{s}-2\lambda^{N}x_{1}(\tilde{v},\tilde{s})=0.$$

This can be rearranged to give


$$x_{1}(\tilde{v},\tilde{s})=x_{2}(\tilde{v},\tilde{s})=\frac{1}{4\lambda^{N}}\tilde{v}-\frac{\mu^{N}}{4\lambda^{N}}\tilde{s}.$$

Therefore

$$\alpha_{0}^{N}=\beta_{0}^{N}=\frac{1}{4\lambda^{N}},$$ (A11)

$$\alpha_{1}^{N}=\beta_{1}^{N}=\frac{\mu^{N}}{4\lambda^{N}}.$$ (A12)

Based on semi-strong efficiency and Lemma 1 and 2 in Zhou [14], it can be concluded that


$$P(\tilde{s},\tilde{v})=E_{k}[\tilde{v}|\tilde{s},\tilde{y}]=E_{k}[\tilde{v}-E_{k}(\tilde{v}|\tilde{s})+E_{k}(\tilde{v}|\tilde{s})|x_{1}(\tilde{v},\tilde{s})+x_{2}(\tilde{v},\tilde{s})+\tilde{u},\tilde{s}]\;=E[\tilde{v}-E_{k}(\tilde{v}|\tilde{s})]+\frac{\mathrm{cov}(\tilde{v}-E_{k}(\tilde{v}|\tilde{s}),2\alpha_{0}^{N}(\tilde{v}-E_{k}(\tilde{v}|\tilde{s}))+\tilde{u})}{\mathrm{var}(2\alpha_{0}^{N}(\tilde{v}-E_{k}(\tilde{v}|\tilde{s}))+\tilde{u})}\cdot \;(2\alpha_{0}^{N}(\tilde{v}-E_{k}(\tilde{v}|\tilde{s}))+\tilde{u})-E[2\alpha_{0}^{N}(\tilde{v}-E_{k}(\tilde{v}|\tilde{s}))+\tilde{u}]+E_{k}(\tilde{v}|\tilde{s})\;=\frac{2\alpha_{0}^{N}H}{{\left(2\alpha_{0}^{N}\right)}^{2}H+\sigma_{u}^{2}}(2\alpha_{0}^{N}(\tilde{v}-E_{k}(\tilde{v}|\tilde{s}))+\tilde{u})+c_{k}\tilde{s}\;=\frac{2\alpha_{0}^{N}H}{(2\alpha_{0}^{N}{)}^{2}H+\sigma_{u}^{2}}\tilde{y}+[\frac{2\alpha_{0}^{N}H}{(2\alpha_{0}^{N}{)}^{2}H+\sigma_{u}^{2}}(-2\alpha_{0}^{N}-2\alpha_{1}^{N})+c_{k}]\tilde{s}.$$

Where

$$c_{k}=\frac{\sigma_{v}^{2}}{\sigma_{v}^{2}+k^{2}\sigma_{\epsilon }^{2}},$$ (A13)

$$H=Var(\tilde{v}-E_{k}(\tilde{v}-\tilde{s}))=E_{k}(\tilde{v}-E_{k}(\tilde{v}|\tilde{s}){)}^{2}=(1-c_{k}{)}^{2}\sigma_{v}^{2}+k^{2}c_{k}^{2}\sigma_{\epsilon }^{2}=\frac{k^{2}\sigma_{v}^{2}\sigma_{\epsilon }^{2}}{\sigma_{v}^{2}+k^{2}\sigma_{\epsilon }^{2}}.$$ (A14)

Therefore

$$\mu^{N}=\frac{2\alpha_{0}^{N}H}{(2\alpha_{0}^{N}{)}^{2}H+\sigma_{u}^{2}}(-2\alpha_{0}^{N}-2\alpha_{1}^{N})+c_{k},$$ (A15)

$$\lambda^{N}=\frac{2\alpha_{0}^{N}H}{(2\alpha_{0}^{N}{)}^{2}H+\sigma_{u}^{2}}.$$ (A16)

Substituting Eqs (41) and (42) into Eq (46) yields


$$\mu^{N}=c_{k}=\frac{\sigma_{v}^{2}}{\sigma_{v}^{2}+k^{2}\sigma_{\epsilon }^{2}}.$$

Substituting $2\alpha_{0}^{N}=\frac{1}{2\lambda^{N}}$ into Eq (46) and simplifying, we can obtain


$$\lambda^{N}=\frac{k\sigma_{v}\sigma_{\varepsilon }}{2\sigma_{u}\sqrt{\sigma_{v}^{2}+k^{2}\sigma_{\varepsilon }^{2}}}.$$

Therefore


$$\alpha_{0}^{N}=\beta_{0}^{N}=\frac{\sigma_{u}\sqrt{\sigma_{v}^{2}+k^{2}\sigma_{\varepsilon }^{2}}}{2k\sigma_{v}\sigma_{\varepsilon }},\;\alpha_{1}^{N}=\beta_{1}^{N}=\frac{-\sigma_{u}\sigma_{v}}{2k\sigma_{\varepsilon }\sqrt{\sigma_{v}^{2}+k^{2}\sigma_{\varepsilon }^{2}}}.\;E[\pi^{N}]=E[(\tilde{v}-P)\;x_{1}(\tilde{v},\tilde{s})]=E[E(\tilde{v}-P)x_{1}(\tilde{v},\tilde{s})|\tilde{v},\tilde{s}]\;=E[(\tilde{v}-\mu^{N}\tilde{s})(\alpha_{0}^{N}\tilde{v}+\alpha_{1}^{N}\tilde{s})-\lambda^{N}(2\alpha_{0}^{N}\tilde{v}+2\alpha_{1}^{N}\tilde{s})(\alpha_{0}^{N}\tilde{v}+\alpha_{1}^{N}\tilde{s})]\;=(\alpha_{0}^{N}+\alpha_{1}^{N}-\alpha_{0}^{N}\mu^{N}-\alpha_{1}^{N}\mu^{N}-2\lambda^{N}(\alpha_{0}^{N}{)}^{2}-4\lambda^{N}\alpha_{0}^{N}\alpha_{1}^{N}-2\lambda^{N}(\alpha_{1}^{N}{)}^{2})\sigma_{v}^{2}\;-(\alpha_{1}^{N}\mu^{N}+2\lambda^{N}(\alpha_{1}^{N}{)}^{2})\sigma_{\epsilon }^{2}.$$

The equation regarding the parameter $\Sigma^{N}$ is


$$\Sigma^{N}=\mathrm{Var}(\tilde{v}|P,\tilde{s})=\mathrm{Var}(\tilde{v}-E(\tilde{v}|\tilde{s})|\lambda^{N}\tilde{y}+\mu^{N}\tilde{s},\tilde{s})\;=\mathrm{Var}(\tilde{v}-E(\tilde{v}|\tilde{s}))-\frac{\mathrm{cov}(\tilde{v}-E(\tilde{v}|\tilde{s}),{2\alpha }_{0}^{N}(\tilde{v}-E(\tilde{v}|\tilde{s}))+\tilde{u}{)}^{2}}{\mathrm{var}({2\alpha }_{0}^{N}(\tilde{v}-E(\tilde{v}|\tilde{s}))+\tilde{u})}\;=\frac{E(\tilde{v}-E(\tilde{v}|\tilde{s}){)}^{2}\sigma_{u}^{2}}{({2\alpha }_{0}^{N}{)}^{2}E(\tilde{v}-E(\tilde{v}|\tilde{s}){)}^{2}+\sigma_{u}^{2}}\;=\frac{\sigma_{v}^{2}\sigma_{\epsilon }^{2}}{\frac{\sigma_{v}^{2}}{k}+\sigma_{v}^{2}+2\sigma_{\epsilon }^{2}}.$$

□

### Appendix A.4. Proof of Proposition 4

***Proof:*** Since the calculation method is similar to Proposition 1, we will briefly summarise the proof process. Let the trading strategy of the insider trader and the pricing rule of the market maker be linear functions:


$$x_{1}(\tilde{s},\tilde{v})=\alpha_{0}^{A}\tilde{v}+\alpha_{1}^{A}\tilde{s}=x_{2}(\tilde{s},\tilde{v})=\beta_{0}^{A}\tilde{v}+\beta_{1}^{A}\tilde{s},P(\tilde{s},\tilde{y})=\lambda^{A}\tilde{y}+\mu^{A}\tilde{s}\text{,}$$

where $\alpha_{0}^{A}$, $\alpha_{1}^{A}$, $\beta_{0}^{A}$, and $\beta_{1}^{A}$ are all constants.

Risk-averse insider traders obtain their trading strategies by maximising the expected value of the negative utility index.


$$E[-e^{-A(\tilde{v}-\tilde{p})x_{1}(\tilde{v},\tilde{s})}|\tilde{s},\tilde{v}]\;=E[-e^{-A(\tilde{v}-\lambda^{A}\tilde{y}-\mu^{A}\tilde{s})x_{1}(\tilde{v},\tilde{s})}|\tilde{s},\tilde{v}]\;=-e^{-A[(\tilde{v}-2\lambda^{A}x_{1}(\tilde{v},\tilde{s})-\mu^{A}\tilde{s})]x_{1}(\tilde{v},\tilde{s})}\cdot E[-e^{-A(-\lambda^{A}\tilde{u})x_{1}(\tilde{v},\tilde{s})}|\tilde{s},\tilde{v}]\;=-e^{-A[(\tilde{v}-2\lambda^{A}x_{1}(\tilde{v},\tilde{s})-\mu^{A}\tilde{s})]x_{1}(\tilde{v},\tilde{s})+\frac{A^{2}(\lambda^{A}{)}^{2}x_{1}(\tilde{v},\tilde{s}{)}^{2}{\sigma_{u}}^{2}}{2}}$$

Taking the first derivative of the above equation and setting it equal to zero, we obtain


$$-e^{-A(\tilde{v}-2\lambda^{A}x_{1}(\tilde{v},\tilde{s})-\mu^{A}\tilde{s})x_{1}(\tilde{v},\tilde{s})+\frac{A^{2}(\lambda^{A}{)}^{2}x_{1}(\tilde{v},\tilde{s}{)}^{2}\sigma_{u}^{2}}{2}}\;\cdot (-A(\tilde{v}-2\lambda^{A}x_{1}(\tilde{v},\tilde{s})-\mu^{A}\tilde{s})+2A\lambda^{A}x_{2}(\tilde{v},\tilde{s})+A^{2}(\lambda^{A}{)}^{2}\sigma_{u}^{2}x_{1}(\tilde{v},\tilde{s}))=0.$$

This can be rearranged to give


$$x_{1}(\tilde{v},\tilde{s})=x_{2}(\tilde{v},\tilde{s})=\frac{1}{4A\lambda^{A}+A^{2}(\lambda^{A}{)}^{2}\sigma_{u}^{2}}\tilde{v}+\frac{-\mu^{A}}{4A\lambda^{A}+A^{2}(\lambda^{A}{)}^{2}\sigma_{u}^{2}}\tilde{s}.$$

Therefore

$$\alpha_{0}^{A}=\beta_{0}^{A}=\frac{1}{4A\lambda^{A}+A^{2}(\lambda^{A}{)}^{2}\sigma_{u}^{2}},$$ (A17)

$$\alpha_{1}^{A}=\beta_{1}^{A}=\frac{-\mu^{A}}{4A\lambda^{A}+A^{2}(\lambda^{A}{)}^{2}\sigma_{u}^{2}}.$$ (A18)

Based on semi-strong efficiency and Lemma 1 and 2 in Zhou [14], it can be concluded that


$$P(\tilde{s},\tilde{v})=E_{k}[\tilde{v}|\tilde{s},\tilde{y}]=E_{k}[\tilde{v}-E_{k}(\tilde{v}|\tilde{s})+E_{k}(\tilde{v}|\tilde{s})|x_{1}(\tilde{v},\tilde{s})+x_{2}(\tilde{v},\tilde{s})+\tilde{u},\tilde{s}]\;=E[\tilde{v}-E_{k}(\tilde{v}|\tilde{s})]+\frac{\mathrm{cov}(\tilde{v}-E_{k}(\tilde{v}|\tilde{s}),2\alpha_{0}^{A}(\tilde{v}-E_{k}(\tilde{v}|\tilde{s}))+\tilde{u})}{\mathrm{var}(2\alpha_{0}^{A}(\tilde{v}-E_{k}(\tilde{v}|\tilde{s}))+\tilde{u})}\cdot \;(2\alpha_{0}^{A}(\tilde{v}-E_{k}(\tilde{v}|\tilde{s}))+\tilde{u})-E[2\alpha_{0}^{A}(\tilde{v}-E_{k}(\tilde{v}|\tilde{s}))+\tilde{u}]+E_{k}(\tilde{v}|\tilde{s})\;=\frac{2\alpha_{0}^{A}H}{{\left(2\alpha_{0}^{A}\right)}^{2}H+\sigma_{u}^{2}}(2\alpha_{0}^{A}(\tilde{v}-E_{k}(\tilde{v}|\tilde{s}))+\tilde{u})+c_{k}\tilde{s}\;=\frac{2\alpha_{0}^{A}H}{(2\alpha_{0}^{A}{)}^{2}H+\sigma_{u}^{2}}\tilde{y}+[\frac{2\alpha_{0}^{A}H}{(2\alpha_{0}^{A}{)}^{2}H+\sigma_{u}^{2}}(-2\alpha_{0}^{A}-2\alpha_{1}^{A})+c_{k}]\tilde{s}.$$

Where

$$c_{k}=\frac{\sigma_{v}^{2}}{\sigma_{v}^{2}+k^{2}\sigma_{\epsilon }^{2}},$$ (A19)

$$H=Var(\tilde{v}-E_{k}(\tilde{v}-\tilde{s}))=E_{k}(\tilde{v}-E_{k}(\tilde{v}|\tilde{s}){)}^{2}=(1-c_{k}{)}^{2}\sigma_{v}^{2}+k^{2}c_{k}^{2}\sigma_{\epsilon }^{2}=\frac{k^{2}\sigma_{v}^{2}\sigma_{\epsilon }^{2}}{\sigma_{v}^{2}+k^{2}\sigma_{\epsilon }^{2}}.$$ (A20)

Therefore

$$\mu^{A}=\frac{2\alpha_{0}^{A}H}{(2\alpha_{0}^{A}{)}^{2}H+\sigma_{u}^{2}}(-2\alpha_{0}^{A}-2\alpha_{1}^{A})+c_{k},$$ (A21)

$$\lambda^{A}=\frac{2\alpha_{0}^{A}H}{(2\alpha_{0}^{A}{)}^{2}H+\sigma_{u}^{2}}.$$ (A22)

Substituting (*A*17) and (*A*18) into (*A*21) yields


$$\mu^{A}=c_{k}=\frac{\sigma_{v}^{2}}{\sigma_{v}^{2}+k^{2}\sigma_{\epsilon }^{2}}.$$

Substituting Eq (47) into Eq (52) and then simplifying, we can obtain a quartic equation involving $\lambda^{A}$.


$$A^{2}\sigma_{u}^{6}\sigma_{v}^{2}(\lambda^{A}{)}^{4}+A^{2}k^{2}\sigma_{u}^{2}\sigma_{\varepsilon }^{2}(\lambda^{A}{)}^{4}+8A\sigma_{u}^{4}\sigma_{v}^{2}{\left(\lambda^{A}\right)}^{3}+8Ak^{2}\sigma_{u}^{4}\sigma_{\varepsilon }^{2}{\left(\lambda^{A}\right)}^{3}\;+16\sigma_{u}^{2}\sigma_{v}^{2}{\left(\lambda^{A}\right)}^{2}+16k^{2}\sigma_{u}^{2}\sigma_{\varepsilon }^{2}{\left(\lambda^{A}\right)}^{2}-2Ak^{2}\sigma_{u}^{2}\sigma_{v}^{2}\sigma_{\varepsilon }^{2}\lambda^{A}-4k^{2}\sigma_{v}^{2}\sigma_{\varepsilon }^{2}=0\text{.}\;E[\pi^{A}]=E[(\tilde{v}-P)\;x_{1}(\tilde{v},\tilde{s})]=E[E(\tilde{v}-P)x_{1}(\tilde{v},\tilde{s})|\tilde{v},\tilde{s}]\;=E[(\tilde{v}-\mu^{A}\tilde{s})(\alpha_{0}^{A}\tilde{v}+\alpha_{1}^{A}\tilde{s})-\lambda^{A}(2\alpha_{0}^{A}\tilde{v}+2\alpha_{1}^{A}\tilde{s})(\alpha_{0}^{A}\tilde{v}+\alpha_{1}^{A}\tilde{s})]\;=(\alpha_{0}^{A}+\alpha_{1}^{A}-\alpha_{0}^{A}\mu^{A}-\alpha_{1}^{A}\mu^{A}-2\lambda^{A}(\alpha_{0}^{A}{)}^{2}-4\lambda^{A}\alpha_{0}^{A}\alpha_{1}^{A}-2\lambda^{A}(\alpha_{1}^{A}{)}^{2})\sigma_{v}^{2}\;-(\alpha_{1}^{A}\mu^{A}+2\lambda^{A}(\alpha_{1}^{A}{)}^{2})\sigma_{\epsilon }^{2}.$$

The equation regarding the parameter $\Sigma^{A}$ is


$$\Sigma^{A}=\mathrm{Var}(\tilde{v}|P,\tilde{s})=\mathrm{Var}(\tilde{v}-E(\tilde{v}|\tilde{s})|\lambda^{A}\tilde{y}+\mu^{A}\tilde{s},\tilde{s})\;=\mathrm{Var}(\tilde{v}-E(\tilde{v}|\tilde{s}))-\frac{\mathrm{cov}(\tilde{v}-E(\tilde{v}|\tilde{s}),{2\alpha }_{0}^{A}(\tilde{v}-E(\tilde{v}|\tilde{s}))+\tilde{u}{)}^{2}}{\mathrm{var}({2\alpha }_{0}^{A}(\tilde{v}-E(\tilde{v}|\tilde{s}))+\tilde{u})}\;=\frac{E(\tilde{v}-E(\tilde{v}|\tilde{s}){)}^{2}\sigma_{u}^{2}}{({2\alpha }_{0}^{A}{)}^{2}E(\tilde{v}-E(\tilde{v}|\tilde{s}){)}^{2}+\sigma_{u}^{2}}\;=\frac{\sigma_{v}^{2}\sigma_{\epsilon }^{2}\sigma_{u}^{2}}{({2\alpha }_{0}^{A}{)}^{2}\sigma_{v}^{2}\sigma_{\epsilon }^{2}+(\sigma_{v}^{2}+\sigma_{\epsilon }^{2})\sigma_{u}^{2}}.$$

□
